# Association of *Escherichia coli *O157:H7 *tir *polymorphisms with human infection

**DOI:** 10.1186/1471-2334-7-98

**Published:** 2007-08-24

**Authors:** James L Bono, James E Keen, Michael L Clawson, Lisa M Durso, Michael P Heaton, William W Laegreid

**Affiliations:** 1 United States Department of Agriculture, Agricultural Research Service, U.S. Meat Animal Research Center, Clay Center, NE, 68933 USA; 2 [Current] Department of Pathobiology, College of Veterinary Medicine, University of Illinois, 2001 South Lincoln Avenue, Urbana, IL 61802

## Abstract

**Background:**

Emerging molecular, animal model and epidemiologic evidence suggests that Shiga-toxigenic *Escherichia coli *O157:H7 (STEC O157) isolates vary in their capacity to cause human infection and disease. The translocated intimin receptor (*tir*) and intimin (*eae*) are virulence factors and bacterial receptor-ligand proteins responsible for tight STEC O157 adherence to intestinal epithelial cells. They represent logical genomic targets to investigate the role of sequence variation in STEC O157 pathogenesis and molecular epidemiology. The purposes of this study were (1) to identify *tir *and *eae *polymorphisms in diverse STEC O157 isolates derived from clinically ill humans and healthy cattle (the dominant zoonotic reservoir) and (2) to test any observed *tir *and *eae *polymorphisms for association with human (vs bovine) isolate source.

**Results:**

Five polymorphisms were identified in a 1,627-bp segment of *tir*. Alleles of two *tir *polymorphisms, *tir *255 T>A and repeat region 1-repeat unit 3 (RR1-RU3, presence or absence) had dissimilar distributions among human and bovine isolates. More than 99% of 108 human isolates possessed the *tir *255 T>A T allele and lacked RR1-RU3. In contrast, the *tir *255 T>A T allele and RR1-RU3 absence were found in 55% and 57%, respectively, of 77 bovine isolates. Both polymorphisms associated strongly with isolate source (p < 0.0001), but not by pulsed field gel electrophoresis type or by *stx*1 and *stx*2 status (as determined by PCR). Two *eae *polymorphisms were identified in a 2,755-bp segment of 44 human and bovine isolates; 42 isolates had identical *eae *sequences. The *eae *polymorphisms did not associate with isolate source.

**Conclusion:**

Polymorphisms in *tir *but not *eae *predict the propensity of STEC O157 isolates to cause human clinical disease. The over-representation of the *tir *255 T>A T allele in human-derived isolates vs the *tir *255 T>A A allele suggests that these isolates have a higher propensity to cause disease. The high frequency of bovine isolates with the A allele suggests a possible bovine ecological niche for this STEC O157 subset.

## Background

Shiga-toxigenic *Escherichia coli *O157:H7 (STEC O157) is the major STEC serotype associated with human infection in the U.S. [1]. Cattle are the predominant North American reservoir of this zoonotic pathogen [2,3] and contact with infected livestock and ingestion of contaminated meat are frequent routes of human infection [4-7]. Other sources of STEC O157 infection are contaminated fruits, vegetables and water [8-10] and person-to-person contact [11,12]. From 1982–2002, there were 350 STEC O157 outbreaks reported in the U.S., resulting in 8,500 clinical cases, 1,500 hospitalizations and 40 deaths [13]. Human STEC O157 infections cause mild self-limiting diarrhea to severe disease including hemorrhagic colitis and hemolytic uremic syndrome (HUS) [14,15]. HUS, due to STEC O157 infection, is the leading cause of renal failure for children under the age of five years [1].

As with many infectious disease agents, STEC O157 strains appear to vary in their capacity to cause human infection and disease. For example, in the gnotobiotic pig challenge model, STEC O157 strains differ in both the clinical course they provoke and the histopathological lesions they induce [16]. Epidemiologic surveillance data in the U.S. also supports the idea of inter-strain variation in STEC O157 virulence. The annual U.S. incidence of clinical STEC O157 infections is estimated at 1.1 per 100,000 persons [1]. However, pooled data from five North American serological surveys found 11% of 2,251 healthy children and adults (11,000 per 100,000 persons) with serologic evidence of *E. coli *O157 exposure and/or subclinical infection [17-21]. On a smaller scale, the investigation of a recent STEC O157 outbreak linked with visiting an agricultural fair suggested that all STEC O157 are not equivalent in terms of their public health risk [22]. At least 25 people out of over 170,000 fair visitors who attended over a two-week period became ill with an STEC O157 isolate that shared the same pulse-field gel electrophoresis (PFGE) pattern. The outbreak investigation revealed that the fairground environment was heavily contaminated with multiple STEC O157 isolates with eight different PFGE patterns, including the outbreak strain. The presumed high human STEC O157 exposure but low human clinical disease incidence, suggested by both the surveillance and outbreak data, could be partially explained if only a subset of STEC O157 isolates present in the bovine (or other) zoonotic reservoirs were pathogenic to humans. Identifying markers for virulent strains as well as understanding the mechanisms responsible for disparities in virulence may provide new insights into the epidemiology and control of STEC O157 infections in both human and animal reservoirs.

An important step in the pathogenesis of human infection with STEC O157 is colonization of the lower gastrointestinal (GI) tract. STEC O157 have a number of virulence and putative virulence factors which aid in this colonization including the locus of enterocyte effacement (LEE), production of Shiga toxin 2, flagellin, OmpA, Lpf and ToxB [14,23-28]. The interaction of two LEE-encoded genes, *tir *and *eae*, is responsible for the tight bacterial adherence to host epithelial cells characteristic of STEC O157 infections. The *eae*-encoded ligand protein intimin is located on the bacterial outer membrane. The intimin receptor protein Tir is translocated into the epithelial cell by type III secretion and integrated in the host cell membrane [29,30]. Given the role of intimin and Tir in STEC O157 pathogenesis and the well documented role of cattle as a zoonotic reservoir, the purpose of this study was to characterize sequence variation in STEC O157 *eae *and *tir *genes and to evaluate whether it associates with human or bovine host origin.

## Methods

### Bacterial strains

For sequence discovery, 22 diverse STEC O157 isolates were assembled that varied by source, either human clinical (n = 9) or bovine (n = 13) (Table 1). A further 101 epidemiologically unrelated human clinical and 64 bovine isolates were included to estimate *tir *polymorphism allele frequencies. Each isolate was characterized by ELISA using anti-O157 and H7 monoclonal antibodies and multiplex PCR for *stx1*, *stx2*, *eae*, *hlyA*, *rfb*_*O157 *_and *fliC*_*H7 *_[31-34]. For the purpose of this study, isolates were defined as STEC O157 if they were *E. coli *O157 antigen positive by ELISA, *rfbE*_O157 _and *fliC*_H7_-positive by PCR, and *stx*1 and/or *stx*2 positive by PCR.

**Table 1:** STEC O157 strains used for sequence polymorphism discovery in the *tir *gene and a representative strain from each *tir *genotype

Strain	H antigen	Collection	Source	Reference	Discovery panel	*tir *genotype^a^
N245	H7	USMARC	Bovine	This paper	N	1
SS NE 1040	H7	USMARC	Bovine	[49]	N	2
CO 50-1	H7	USMARC	Bovine	[49]	Y	3
NE 972-1	H7	USMARC	Bovine	[49]	Y	3
KS 368-1	H7	USMARC	Bovine	[49]	Y	4
KS 546-2	H7	USMARC	Bovine	[49]	Y	4
NE 1370-3	H7	USMARC	Bovine	[49]	Y	4
TX 265-1	H7	USMARC	Bovine	[49]	Y	4
TX 376-2	H7	USMARC	Bovine	[49]	Y	4
TX 723-1	H7	USMARC	Bovine	[49]	Y	4
CO 147-1	H7	USMARC	Bovine	[49]	Y	4
NE 1127-1	H7	USMARC	Bovine	[49]	Y	5
TW07587	H7	EHEC 1–3	Human	[50]	Y	5
2	NM	Acheson	Human	^b^	Y	5
USDA81	H7	CDC	Human	This paper	N	6
CO 713-5	H7	USMARC	Bovine	[49]	Y	7
MARC 611	H7	USMARC	Bovine	[49]	Y	7
NE 1270-2	H7	USMARC	Bovine	[49]	Y	7
31277	H7	IDPH	Human	^c^	Y	7
1	H7	Mandrell	Human	^d^	Y	7
35150	H7	ATCC	Human	ATCC	Y	7
43889	H7	ATCC	Human	ATCC	Y	7
43890	H7	ATCC	Human	ATCC	Y	7
Sakai	H7	RMID	Human	[45]	Y	7
TW04863	H7	EHEC 1–2	Human	[50]	Y	7
N4009-114-4	H7	APHIS	Bovine	This paper	N	8
TW06555	H7	EHEC 1–6	Human	[50]	N	9
TW00116	H7	EHEC 1–4	Human	[50]	N	10

^a ^As described in Figure 1B.

^b ^Dr. David Acheson, DHHS, FDA, College Park, MD.

^c ^Enteric Lab, Illinois Department of Public Health.

^d ^Dr. Robert Mandrell, USDA, ARS, Albany, CA.

### PCR amplification, DNA sequencing and analysis

A 2,755-kb segment of the *eae *gene and 1,627-kb segment of the *tir *gene were amplified and sequenced using primers listed in Table 2. The amplification reactions contained 0.5 ng of DNA, 0.75 uM of each primer, 200 uM of each dNTP, 1.5 mM MgCl_2 _and 1 U of Platinum Taq DNA polymerase (Invitrogen Corporation, Carlsbad, CA) in a 55 ul reaction. PCR amplifications were performed using a PTC-200 (MJ Research, Waterton, MA) at the following conditions: 1 min at 95°C followed by 30 sec at 96°C, 30 sec at 52°C and 2 min at 72°C for 35 cycles and finally 72°C for 7 min for 1 cycle.

**Table 2:** Oligonucleotides (5'-3') used in this study

Oligonucleotide	Sequence	Orientation^a^	Application
tir:1U19	atg cct att ggt aat ctt g	Sense	*tir *5' amplification sequencing
tir:72U17	aca aac cga cgg tgc ag	Sense	*tir *sequencing
tir:473L17	atg ccc agc acc acc ac	Antisense	*tir *sequencing
tir:521U17	aag ccc gcc aaa gga ta	Sense	*tir *RR1 sequencing
tir:967L17	tct tcg cct gct gct tt	Antisense	*tir *RR1 sequencing
tir:1046U17	cta aac gcc agg agg ag	Sense	*tir *RR2, 3, & 4 sequencing
tir:1572L17	ggc gta atc cac cac tt	Antisense	*tir *RR2, 3, & 4 sequencing
tir:1638L16	cgc tgg tgg gtt att c	Antisense	*tir *3' amplification sequencing
eae:16U16	tgt tat acc cgg acc c	Sense	*eae *5' amplification sequencing
eae:135U19	taa att ggg ttc gga ttc a	Sense	*eae *sequencing
eae:382L17	aca aga ggt gcc gaa cc	Antisense	*eae *sequencing
eae:490U16	aat tat gcg gca caa c	Sense	*eae *sequencing
eae:574L18	gtt acc agc gat acc aag	Antisense	*eae *sequencing
eae:626U18	att atg gaa cgg cag agg	Sense	*eae *sequencing
eae:886L19	ttt tga aat agt ctc gcc a	Antisense	*eae *sequencing
eae:1090U17	gat aag ctg cag tcg aa	Sense	*eae *sequencing
eae:1178L18	ttt cat tac ccg tac cat	Antisense	*eae *sequencing
eae:1290U18	atc agg cag ccg tta cga	Sense	*eae *sequencing
eae:1492L19	ttc cgc tat gct gaa tct g	Antisense	*eae *sequencing
eae:1695U18	gga taa gac ttc ggc taa	Sense	*eae *sequencing
eae:1732L18	cgt cgc ggt ata agt aat	Antisense	*eae *sequencing
eae:1883U17	cgc cag gac agg tcg tc	Sense	*eae *sequencing
eae:2135L17	att tcc cgt ggt tgc tt	Antisense	*eae *sequencing
eae:2249U19	ttg gta aca atg tca gag g	Sense	*eae *sequencing
eae:2358L13	acc acc gct tgc t	Antisense	*eae *sequencing
eae:2360U17	caa gcg gtg gtg atg gt	Sense	*eae *sequencing
eae:2699L17	tcc aga acg ctg ctc ac	Antisense	*eae *sequencing
eae:2787L19	tta ttc tac aca aac cgc a	Antisense	*eae *3' amplification sequencing

^a ^With respect to *tir *and *eae *mRNA.

PCR products were purified and concentrated using the QIAquick PCR Purification Kit (Qiagen Inc., Valencia, CA). DNA sequencing reactions were prepared using the ABI PRISM BigDye terminator cycle sequencing ready reaction kit (PE Applied Biosystems, Foster City, CA) with slight modifications of the manufacturer's protocol to reduce the final volume to 10 ul. The sequencing reactions were cycled with a PTC-200 (MJ Research) at the following conditions: 1 min at 96°C followed by 30 sec at 96°C, 1 min at 50°C and 4 min at 60°C for 30 cycles. DNA sequences were determined with either an ABI PRISM 3700 DNA analyzer or an ABI PRISM 377 DNA sequencer (PE Applied Biosystems).

Nucleotide sequences were analyzed using SeqMan and alignments were constructed using Clustal X, both from the Lasergene software package (DNASTAR, Inc., Madison, WI). A consensus parsimony tree was generated in PHYLIP (version 3.65) from *tir *DNA sequences using the program PARS [35] and viewed in TreeView (version 1.6.6) [36].

### Pulse field gel electrophoresis of isolates used for genotyping

PFGE was performed on all human and bovine derived *E. coli *O157:H7 isolates by using the PulseNet protocol and the restriction endonuclease *Xba*I [37]. Restriction fragment patterns were analyzed using Bionumerics version 4 (Applied Maths, Belgium).

### Statistical analysis of *tir *variation and host association

The frequencies of each identified *tir *nucleotide or repeat polymorphism and *tir *genotype were compared between STEC O157 isolates of human and bovine origin. The data were analyzed as an unmatched case-control study by exact logistic regression using the LOGISTIC procedure of SAS 9.1 (SAS Institute, Inc., Cary, NC). The binary response variable (outcome) of interest was the probability of the strain being of bovine origin (case) vs human origin (control). Each *tir *polymorphism and genotype was converted into a categorical explanatory (predictor) variable, where each possible variant within a given polymorphism was coded separately. Genotype 10 and the most common variant for each polymorphism were used as reference conditions. The association of each *tir *polymorphism variant with the likelihood of being a case STEC O157 strain was examined by generating univariate exact odds ratios (OR) with exact 95% confidence intervals (CI) and corresponding p values. *Stx *profiles of isolates defined by PCR were also examined for association with human or bovine strain origin.

## Results

### Polymorphisms in STEC O157 *tir *and *eae*

**Figure 1:**
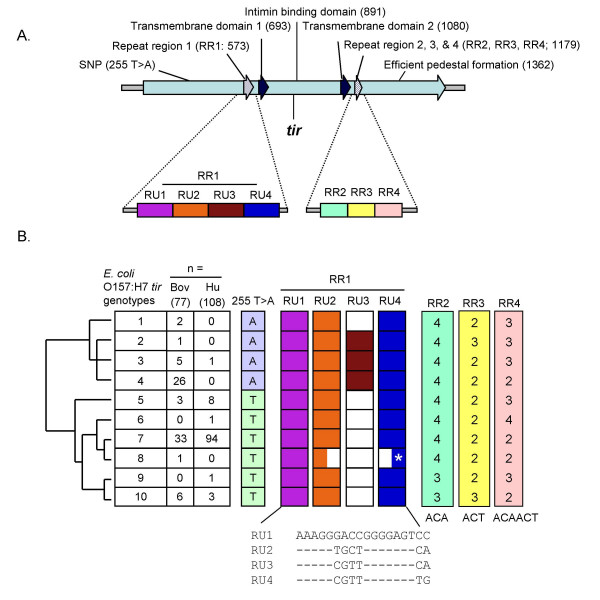
**Physical maps and taxonomical relationships of the *tir *gene**. (A) A physical map of the *tir *gene from STEC O157 (GenBank accession number BA000007 gene ECs4561). A 1.6-kb region of the *tir *gene was sequenced from 22 STEC O157 isolates and the polymorphisms mapped on the *tir *gene along with previously identified functional domains. (B) Consensus tree from seven equally parsimonious trees constructed in PHYLIP (version 3.65) [35] with the program PARS and viewed in TreeView (version 1.6.6) [36]. The tree was generated from seven variable sites (255 T>A, RR1-RU2, RR1-RU3, RR1-RU4, RR2, RR3 and RR4) and describes the taxonomical relationship of the *tir *nucleotide sequence with associated phenotypic and genotypic information. Ten genotypes resulting from one nucleotide polymorphism and four repeat region variations were identified from the 185 isolates. Colored boxes identify the different nucleotide variations and repeat units, while white boxes indicate missing repeat units. The sequences of the repeat regions are located below the colored boxes with the top line as the consensus sequence. A variant repeat unit (*) in repeat region 1 was identified that had the following sequence "AAAGGTGCTGGGGAGTTG".

Five polymorphic loci were identified in a 1,627-kb segment of the STEC O157 *tir *gene [GenBank:DQ458771]. One was a single nucleotide polymorphism (SNP), 255 T>A (reference sequence is GenBank accession number BA000007 gene ECs4561). The minor allele of this non-synonymous polymorphism encodes aspartic acid while the other encodes glutamic acid. Four were repeat polymorphisms with the following properties: repeat region 1 (RR1, nucleotide position 573 in reference sequence BAB37984) containing up to four imperfect 18-bp repeat units (RU1-RU4), RR2 (nucleotide position 963) containing [ACA]_n _where n = 3 or 4, RR3 (nucleotide position 1,080) containing [ACT]_n _where n = 2 or 3 and RR4 (nucleotide position 1,179) containing [ACAACT]_n _where n = 2, 3 or 4 (Figure 1A). In one isolate, a chimeric repeat within RR1 was identified consisting of approximately the 5' half of RU2 and the 3' half of RU4 (Figure 1B).

The five polymorphic *tir *loci defined ten unique *tir *genotypes (Figure 1B). Two genotypes, 4 and 7, accounted for 83% (n = 185) of the isolates sequenced, while four genotypes were observed in only one isolate each (Figure 1B). A consensus parsimony tree generated from these genotypes defined two major clades (Figure 1B). Alleles of *tir *255 T>A and RR1-RU3 were responsible for discrimination between these clades (Figure 1B). Alleles of these two polymorphisms are strongly correlated.

The sequence of a 2,755-bp region of the *eae *gene was identical in 42 of 44 STEC O157 isolates representing at least one isolate from each *tir *genotype [GenBank:EF540940]. Synonymous (*eae *60 G>A reference sequence is GenBank accession number BA000007 gene ECs4559) and non-synonymous (*eae *2414 C>T) SNPs were identified, each in isolates 2857\98 [GenBank:EF540939] and ATCC 43890 [GenBank:EF540941], respectively. The *eae *2414 C>T results in a change from a threonine to an isoleucine (T805I). This isolate also contained *tir *genotype 7, the most common *tir *genotype.

### Association of *tir *255 T>A and RR1-RU3 alleles with host origin of STEC O157 isolates

Unmatched case-control analysis showed that only *tir *255 T>A and RR1-RU3 (presence or absence) alleles were significantly associated with host origin. Specifically, isolates with *tir *255 T>A A allele were 34.0 times more likely (5.7 to 1381.9 95% CI, p < 0.0001) to be of bovine than human origin. Similarly, isolates with RR1-RU3 present were 32.0 times more likely (5.3 to 1302.9 95% CI, p < 0.0001) to be of bovine than human origin. Because *tir *255 T>A and RR1-RU3 alleles discriminate between genotypes 4 and 7, these genotypes also associate with host origin. Specifically, isolates with genotype 4 were 37.0 times more likely to be of bovine than human origin (6.6 to ∞ 95% CI, p < 0.0001), while those with genotype 7 were 2.9 times more likely to be of human than bovine origin (2.0 to 4.5 95% CI, p < 0.0001). Genotyping of 255 T>A on all study isolates reinforced this association, with 1 out of 108 human isolates (0.93%, 0.02 to 5.1 95% CI), as opposed to 34 out of 77 bovine isolates (44.2%, 32.8 to 55.9 95% CI), having an A at 255 T>A (p < 0.0001). Furthermore, *stx*1 and *stx*2 status (as determined by PCR) were statistically independent of bovine or human derived STEC O157 isolates (p = 0.12 and p = 0.37, respectively) and when all 185 STEC O157 isolates were compared by PFGE, there was no association of PFGE profiles with *tir *255 T>A alleles.

## Discussion

STEC O157 *tir *255 T>A T allele is significantly overrepresented in human isolates relative to isolates with the A allele (99.1 vs. 0.9%, p < 0.0001). The reason for this host difference in 255 T>A allele frequency is unknown. It is possible that STEC O157 255 T>A A isolates are shed in cattle feces for shorter periods of time or in lower numbers than STEC O157 255 T>A T isolates, resulting in lower probability of product or environmental contamination and thus, lower human exposure and subsequent infection. Reduced human exposure could also occur if STEC O157 255 T>A A isolates are less able to survive on meat, on produce, in water or in other environmental sources of human infection. STEC O157 255 T>A A isolates maybe less virulent in humans so that they are less likely to cause clinical illness prompting fecal culture. Whatever the actual mechanism, the low 255 T>A A allele frequency in human isolates cannot be explained by a corresponding low frequency in the bovine reservoir.

Non-random distribution of *E. coli *O157:H7 subtypes among bovine and human isolates have been reported in previous investigations. Octamer-based genome scanning classified STEC O157 into two lineages (I and II), with human origin isolates biased towards lineage I [38]. Q-gene allelic variation (upstream of the prophage *stx *region), Shiga-toxin 2 production differences and Shiga toxin-encoding bacteriophage insertion site-defined genotypes also had biased distributions of isolates from bovine and human origin [39-41]. However, none of these previously described methods provided as clear a discrimination between human and bovine isolates as those described in this study. Furthermore, the presence of one or both *stx*1 and *stx*2 genes (as determined by PCR) was statistically independent of an isolate's *tir *255 T>A allele or RU3 presence or absence. The high degree of discrimination provided by *tir *255 T>A and the central role of Tir in human infection points towards a possible functional role, rather than solely as a marker, for this polymorphism.

Previous studies indicate a paucity of nucleotide polymorphisms in most STEC O157 genes [42-44]. The presence of five polymorphic loci with high minor allele frequency within *tir*, therefore, appears to be atypical in STEC O157. Furthermore, all five polymorphisms are non-synonymous. In contrast, only one low frequency synonymous polymorphism was found in *eae*, suggesting that these two loci are under different selective pressures. The association of *tir *255 T>A T allele with human infection argues that host factors may impose some selection pressure on *tir*. The fact that the *tir *255 T>A A minor allele frequency is over 30% in bovine isolates, where the frequency of minor alleles for most SNPs in STEC O157 is considerably less than that, also argues for some selection on this allele or another locus tightly linked to 255 T>A [43,44].

Limited information exists on complete *tir *gene sequence from STEC O157. Examination of the two published STEC O157 genomic sequences, EDL 933 and Sakai [45,46], showed that their *tir *genes were both genotype 7. Our sequencing of the *tir *gene from these two isolates confirmed this finding (data not shown for EDL 933). RR2, RR3 and RR4 together were previously used as a marker for high-resolution molecular typing of *E. coli *O157:H7 [47]. In the present study, the sequencing of a broad population of STEC O157 strains that included both human clinical and bovine reservoir isolates revealed additional informative *tir *polymorphisms, particularly the 255 T>A A allele and the presence of RR1-RU3.

Two polymorphic *tir *loci, *tir *255 T>A and RR1-RU3, appear to have epidemiologic significance by their clear and strong association with isolate host source. Both loci are located near the amino terminus of Tir, a portion of the molecule that is normally located in the host cytosol during Tir-Intimin binding in host cell-bacterial adherence [48], in a region where no function has been described. However, these loci may have functional significance based on the biased distribution of their alleles in human derived isolates. One explanation for this could be variation in avidity, kinetics or tropism of adherence to epithelial cells, a major function of the Tir protein, from isolates with the *tir *255 T>A A allele compared to isolates with the *tir *255 T>A T allele. However, more investigation will be necessary to delineate the structure-function relationships of these *tir *polymorphisms.

## Conclusion

Many host, bacterial and environmental factors impact whether or not infection results from human exposure to STEC O157. This study demonstrates that genomic polymorphisms in *tir *but not *eae *predict the likelihood that STEC O157 strains can cause human disease. Intriguing but unexplained findings include the host bias in *tir *allele frequency between human and bovine hosts. The over-representation of the *tir *255 T>A T allele in human-derived isolates – vs the A allele proves its merit as a marker for virulence in humans. Also of interest is the high degree of *tir *sequence variation relative to that found in *eae*, even though the two proteins encoded by these genes interact together as receptor and ligand during adherence to host intestinal epithelial cells. Further research will be necessary to determine if the *tir *255 T>A and RR1-RU3 polymorphisms are simply markers of strain virulence or are functional components of host-pathogen interactions.

## Competing interests

The authors declare that they have no competing interests.

## Authors' contributions

JLB co-conceive the study and experimental design, carried out the DNA sequencing and editing of the *tir *and *eae *genes and drafted the manuscript. JEK provided isolates for the study, carried out the statistical analysis and helped edit the manuscript. MLC carried out the phylogenic analysis of the *tir *DNA sequence. LMD participated in the PFGE analysis of the isolates and helped edit the manuscript. MPH participated in DNA sequence analysis. WWL co-conceived the study and experimental design, carried out the initial sequencing of the *tir *and *eae *genes and helped draft and edit the manuscript. All authors read and approved the final manuscript.

## Pre-publication history

The pre-publication history for this paper can be accessed here:


